# Effects of early- and mid-life stress on DNA methylation of genes associated with subclinical cardiovascular disease and cognitive impairment: a systematic review

**DOI:** 10.1186/s12881-019-0764-4

**Published:** 2019-03-12

**Authors:** Elena M. Vidrascu, Alexander C. Bashore, Timothy D. Howard, Justin B. Moore

**Affiliations:** 1 Department of Family & Community Medicine, Wake Forest School of Medicine, Winston-Salem, NC USA; 2 Department of Internal Medicine, Wake Forest School of Medicine, Winston-Salem, NC USA; 3 Department of Biochemistry, Wake Forest School of Medicine, Winston-Salem, NC USA; 4 Department of Epidemiology and Prevention, Wake Forest School of Medicine, Winston-Salem, NC USA

**Keywords:** Cardiovascular disease, Cognitive impairment, DNA methylation, Early-life stress, Epigenetics, Mid-life stress, Stress exposure

## Abstract

**Background:**

Traditional and novel risk factors cannot sufficiently explain the differential susceptibility to cardiovascular disease (CVD). Epigenetics may serve to partially explain this residual disparity, with life course stressors shown to modify methylation of genes implicated in various diseases. Subclinical CVD is often comorbid with cognitive impairment (CI), which warrants research into the identification of common genes for both conditions.

**Methods:**

We conducted a systematic review of the existing literature to identify studies depicting the relationship between life course stressors, DNA methylation, subclinical CVD, and cognition.

**Results:**

A total of 16 articles (8 human and 8 animal) were identified, with the earliest published in 2008. Four genes (*COMT*, *NOS3*, *Igfl1*, and *Sod2*) were analyzed by more than one study, but not in association with both CVD and CI. One gene (*NR3C1*) was associated with both outcomes, albeit not within the same study. There was some consistency among studies with markers used for subclinical CVD and cognition, but considerable variability in stress exposure (especially in human studies), cell type/tissue of interest, method for detection of DNA methylation, and risk factors. Racial and ethnic differences were not considered, but analysis of sex in one human study found statistically significant differentially methylated X-linked loci associated with attention and intelligence.

**Conclusions:**

This review suggests the need for additional studies to implement more comprehensive and methodologically rigorous study designs that can better identify epigenetic biomarkers to differentiate individuals vulnerable to both subclinical CVD and associated CI.

**Electronic supplementary material:**

The online version of this article (10.1186/s12881-019-0764-4) contains supplementary material, which is available to authorized users.

## Background

Cardiovascular disease (CVD) is the leading cause of death in the United States, with more than an estimated 1 in 3 Americans diagnosed with at least one type of CVD [1]. Epidemiologic studies suggest a strong association between CVD and type 2 diabetes mellitus (T2DM), the latter of which affects over 25 million Americans [2]. The Diabetes Heart Study (DHS) contains a large cohort of these dual diagnosis individuals, and follow-up studies done with this population have found markers of subclinical CVD as predictors of cognitive function in diabetic patients, warranting early intervention to prevent CVD and related cognitive deficits [3]. Independent of T2DM, CVD and cognitive impairment (CI) are frequently co-occurring conditions, with empirical research supporting the association between subclinical CVD markers and dysfunction in cognitive domains such as memory, attention, and decision making [3–8]. Markers such as atrial fibrillation and coronary artery calcium may also serve as risk factors for future cognitive decline and dementia [5, 9, 10]. In a similar fashion, the degree of CI can predict the risk and severity of CVD events and mortality [6, 7]. Efforts aimed at identifying commonalities between subclinical CVD and cognition may serve as one approach to prevent clinical diagnosis of CVD and cognitive decline, which is especially prevalent in individuals with T2DM.

Several traditional and novel risk factors have been reported for CVD. Traditional ones include family history, smoking, lipid levels, diabetes mellitus, physical activity, blood pressure, and stress [11]. There may be similar overlap between CVD risk factors and risk for cognitive decline [12]. Novel risk factors for CVD include increased levels of inflammatory markers such as C-reactive protein and interleukin-6 [13]. Sex, race, and ethnicity also serve as potential moderators, with greater CVD prevalence among males than females, and greater CVD-related death rates among non-Hispanic blacks than non-Hispanic whites. However, there is still much variability, warranting research into the mechanisms that can explain residual disparity among individuals.

**Fig. 1:**
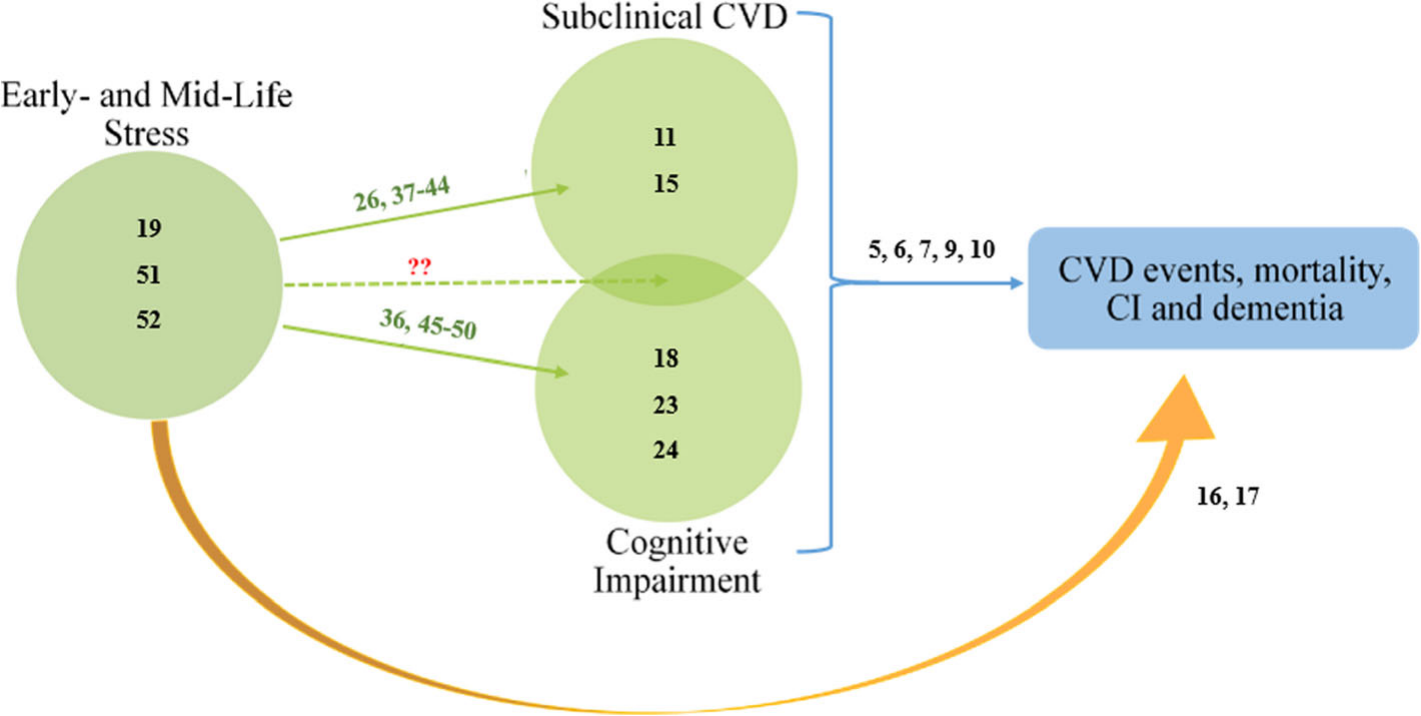
Relationship between early- and mid-life stress and methylated genes associated with adverse health. Blue arrow represents the latter relationship and studies supporting this are depicted by black numbers. Orange arrow represents the relationship between life stress and future CVD events, mortality, CI and dementia, with black numbers representing studies supporting this relationship. Black numbers within green circles represent studies which have analyzed methylated genes within that category alone. Green arrows represent the relationship between life stress and methylated genes implicated in (top) subclinical CVD; (bottom) CI; and (middle) both subclinical CVD and CI. Studies analyzed in this systematic review which illustrate these relationships are depicted by green numbers. Red question marks illustrate no studies found to support this relationship. Numbered studies can be found in the References section

Stress (i.e. job strain, parental loss of child, illness diagnosis) is a risk factor for both CVD and CI, with increased risk of coronary heart disease mortality in those who experienced three or more stressful life events [14]. Exposure to stress can modify DNA methylation, which may alter gene expression and therefore contribute to disease phenotypes [15]. Early-life stress, such as childhood abuse and stress-related disorders, have lasting effects on methylation that may persist into adulthood [16–19]. Childhood adversities and stress at other points throughout the lifespan may induce health problems by dysregulating physiological systems like the immune, nervous, metabolic, and neuroendocrine systems. The broad and often subjective nature of stress makes it difficult to accurately measure, which is further complicated when considering temporal differences in stress exposure: acute and chronic stressors have different effects on health. Whether it be from a single experience or an accumulation of experiences, greater stress duration can contribute to more toxic and long-term changes in physiological responses in contrast to acute events [20, 21]. Since DNA methylation may mediate the effects of stress on susceptibility to CVD and CI, it is paramount that studies comprehensively gauge stress. There is a lack of research identifying any shared genetic risk factors for clinical CVD and CI, with CI being independent of a neurological disorder [22], but recent research has focused on the identification of CpG sites specifically methylated in genes that are associated with subclinical CVD and CI [15, 23, 24]; however, there is great inconsistency with methods used to identify and quantify methylation. To our knowledge, no review elucidates whether stress can act to modify the methylation status of genes implicated in both subclinical CVD and CI, and therefore contribute to future manifestation of disease. Toward this end, we conducted a systematic review of the literature investigating these relationships (Fig. 1).

## Methods

### Study identification

The reporting in this systematic review is consistent with the Preferred Reporting Items for Systematic Reviews and Meta-Analyses (PRISMA) statement (Additional file 1: Table S1) [25]. The Cochrane Database of Systemic Reviews was checked to rule out any possibility of another review conducted on a similar topic. PubMed was the only database used to retrieve studies. Due to the lack of standardization among studies in this review (explained further in Discussion), a meta-analysis was not feasible. Our searches were not limited by year of publication since the study of epigenetics as a contributor to complex diseases is relatively new. All included studies were published within the last six years, with the exception of one published in 2008 [26]. Only articles in English and those investigating humans or other mammals were included. Animal studies were included so as to compare methylation differences observed between similar stress exposures (with humans). Subclinical CVD and cognitive impairment were not limited to any particular conditions due to the already narrowed focus of the research question and is reflected by the broad use of terms used in the search strategy. Clinical CI and CVD were excluded so that only preclinical risk factors were captured. Subclinical CVD terms are common clinical markers for CVD and were abstracted from review papers and papers cited within those papers [3, 5, 27–29]. CI terms were those that related to cognition, without regard for any specific condition. All terms were refined using MeSH categories in the Pubmed database. A complete list of the combination of methylation terms AND either cognitive impairment OR subclinical cardiovascular disease terms is shown in Additional file 2.

### Study selection

With the intent to include a broad spectrum of stress exposures, terms related to stress were not specified in the search and instead studies were screened based on certain selection criteria (Additional file 3: Tables S2-S3). The primary requirement was that direct exposure to stress be experienced postnatally, from early- to mid-life. To remain within the scope of this review, parental and prenatal stress exposures were excluded. If there is a parental stressor that also affects the child/pup postnatally (e.g. maternal separation), then this is included. We defined our maximum age for mid-life to be 50 years for humans and 8 months for animals, at which point the animals would have reached social maturity [30]. We included illness as a stressor if onset occurred prior to the ages defined as mid-life. Living with chronic illness comes with emotional and physical daily stressors, contributing to varying amounts of subjective stress among individuals [14]. With the already limited scope of studies expected to be within the literature that pertained to our research question, we wished to keep the stress requirement broad. Although there may be biological effects of the illness itself that could confound any results observed and thus make it difficult to parse out what contributes to changes in DNA methylation, the key interest is consistent DNA methylation patterns among studies. Environmental chemical exposure was excluded as a stressor due to the unlikely possibility that an affected individual would have any subjective stress directly related to the awareness of any chemical exposure. Studies with the CI outcome as a symptom of a specific disease (e.g. Alzheimer’s) were excluded so that results could be applicable to the general population. Our initial search retrieved 902 articles. The first and second authors screened the titles and abstracts of all articles based on the following criteria: (1) evidence of quantitatively measured DNA methylation; (2) evidence of early- or mid-life stress; and (3) evidence of either subclinical CVD or CI. A total of 50 studies remained for further full-text screening, 14 of which were included for narrative analysis. The authors screened the bibliographic references of full-text studies, giving a final total of 16 studies. A flow diagram of this process and details on excluded studies are available in Additional file 4: Figure S1 and Additional file 3: Tables S2-S3.

### Data extraction

General characteristics of human studies were extracted based on eight variables: 1) study design; 2) location of study; 3) sample size; 4) sex; 5) age; 6) risk factors; 7) outcome(s); and 8) quality of study (Additional files 5-6: Tables S4-S5). General characteristics of animal studies were extracted based on five variables: 1) experimental group; 2) sample size; 3) sex; 4) age at outcome assessment; and 5) outcome(s) (Additional file 7: Tables S6-S7). Information on stress exposure, outcome, and DNA methylation from each study was extracted based on six variables: 1) exposure; 2) outcome; 3) gene(s) with greatest association; 4) regions analyzed for methylation 5) methylation method; and 6) summary of associations (Tables 1 and 2). Information on human- and animal-based studies was analyzed separately.

**Table 1:** Associations between DNA methylation and subclinical cardiovascular disease

	Lead Author, Publication Date	Exposure	Outcome	Gene (s) with Greatest Association	Regions Analyzed for Methylation	Methylation Method	Summary of Correlations
Human Studies	Chen et al. 2016^39^	Obstructive sleep apnea	Hypertension^s^	ASF1A, KLHL28^*^, CCL26, CNR1, CTSZ, CXCR3, FLJ46358, GIMAP5, HOXB2, IL1R2, LTA, TMEM175^*^, OFD1, OR10H2, OR1G1, ORM1, PTCD2, SP140, TRHR, TRPM2, PRSS1^*^, NRSN1^*^	Promoter regions analyzed in 22 genes	HumanMethylation27 Bead Chip and Pyrosequencing	Both ↑ and ↓ methylation in 22 genes associated with ↑ hypertension
	Kheirandish-Gozal et al. 2013^38^	Obstructive sleep apnea	Endothelial dysfunction (post occlusive hyperemic response)	NOS3^**^	Proximal promoter region analyzed	Pyrosequencing	↑ methylation at CpG site (− 171 bp from TSS) correlated with ↑ endothelial dysfunction
	Nanayakkara et al. 2008^26^	Stage 2–4 chronic kidney disease	Atherosclerosis (CCA-IMT) Endothelial dysfunction (BA-FMD)	**–**	Genome-wide	Liquid chromatography-tandem mass spectrometry (LC-MS/MS)	No correlation between methylation and either atherosclerosis or endothelial dysfunction
	Zhao et al. 2015^37^	Vietnam war veteran	Endothelial dysfunction (BA-FMD)	NR3C1	22 CpG sites analyzed within exon 1_F_ promoter region	Pyrosequencing	↑ methylation at 12 CpG residues associated with ↑ endothelial dysfunction
Animal Studies	Chu et al. 2015^44^	Intermittent hypoxia	Endothelial dysfunction (post ischemic reperfusion response) Hypertension (systolic blood pressure)	Ace***, Agt	CpG island surrounding TSS of *Ace* gene analyzed Regulatory region 3000 bp upstream of TSS of *Agt* gene analyzed	DNA methylation-sensitive enzymatic restriction and quantitative PCR	Trend towards ↓ methylation at a CpG island in *Ace* TSS and upstream of *Agt* TSS in association with ↑ endothelial dysfunction and ↑ hypertension
	Nanduri et al. 2012^43^	Intermittent hypoxia	Cardio-respiratory dysfunction (breathing, blood pressure)	Sod2	–	Bisulfite sequencing	↑ methylation at a CpG site in the first intron (+ 128 bp from TSS) associated with ↑ cardio-respiratory dysfunction
	Nanduri et al. 2017^40^	Intermittent hypoxia	Hypertension (blood pressure)	Prdx4, Sod1, Sod2, Txnrd2	–	Bisulfite sequencing and Epitect Methyl II custom PCR array	↑ methylation in 3 genes and at CpG region 4 (+ 157 bp from TSS) of *Sod2* associated with ↑ blood pressure
	Yang et al. 2015^41^	Normobaric hypoxia	Pulmonary hypertension (RVSP, PVR, RVH)	Igfl1	CpG sites around promoter region analyzed	Amplicon sequencing	↑ methylation at 1 out of 5 CpG sites around promoter region associated with ↑ pulmonary hypertension; ↑ global DNA methylation after 2 weeks of chronic hypoxia
	Zhang et al. 2014^42^	Extrauterine growth restriction	Pulmonary hypertension (RVSP, RVH, PVR, PAP)	Nos3**, Fgfr2, Igfl1, Med1****, Rhoc, Notch1	DML withinpromoter region and CpG islands analyzed	MeDIP-chip microarray	↑ methylation within promoter of *Fgfr2*, *Rhoc*, *Notch1*, and *Igf1* associated with development of hypertension; no difference in total methylation within *Nos3* and *Med1* promoter regions

^s^self-report

*reported as BTBD5, MGC4618, TRY1, and VMP, respectively, in Chen et al. 2016 study; **reported as eNOS in Kheirandish-Gozal et al. 2013 and Zhang et al. 2014 studies; ***reported as Ace1 in Chu et al. 2015 study; ****reported as Ppar in Zhang et al. 2014 study

Note: complete list of identified genes is not included for study Chen et al. 2016

CVD cardiovascular disease; BA-FMD brachial artery flow-mediated dilatation; CCA-IMT common carotid artery intima-media thickness; NA non-applicable; PAP pulmonary artery pressure; PVR pulmonary vascular remodeling, RVH right ventricular hypertrophy; RVSP right ventricular systolic pressure; TSS transcription start site

**Table 2:** Associations between DNA methylation and cognitive impairment

	Lead Author, Publication Date	Exposure	Outcome	Gene (s) with Greatest Association	Regions Analyzed for Methylation	Methylation Assessment	Summary of Correlations
Human Studies	Alelu-Paz et al. 2015^36^	Schizophrenia	Memory (MIS), global executive function (FAB), cognitive mental state (MMSE)	DISC1, DRD1, GABBR2, HINT1, MB-COMT, RELN, SLC6A4	CpG islands within promoter region of 19 genes analyzed	Methylation-specific PCR and bisulfite genomic sequencing	No methylated CpGs, regardless of severity of cognitive impairment
	Levine et al. 2016^46^	HIV	Learning, memory, attention, and motor (battery of tests)	**–**	Genome-wide	HumanMethylation450 Bead Chip	Significant positive association between DNA methylation age and diagnosis of HAND
	Peter et al. 2016^45^	Postnatal malnourishment	Attention (CAARS)	ABCF1, COMT, DCTN1-AS1, FOXP2, IFNG, INHBB, MIR200B, RAB3B, SYNGAP1, VARS, VIPR2, WT1	Genome-wide	HumanMethylation450 Bead Chip	↑ methylation in 609 and ↓ methylation in 391 CpG sites in autosomal DMRs; < 13 DMRs moderately correlated w/ADHD index; 15 X-linked DMR in males, and 1 in females, associated with attention
			Intelligence (WASI)	SYNGAP1, ZBTB9			< 3 sites significantly associated with IQ scores; 15 X-linked DMR in males, and 1 in females, associated with IQ scores
	Ursini et al. 2011^47^	Obstetric complications and/or stressful life events^s^	Working memory (*n*-back task)	COMT	CpG sites at rs4680 site (C2) analyzed 3 CpG sites within gene promoter analyzed	Pyrosequencing	C2 methylation of *COMT* promoter in Val/Val negatively correlated with stress and positively correlated with working memory accuracy
Animal Studies	Cordner et al. 2016^48^	Chronic variable stress	Recognition memory (NOR) Spatial learning and memory (Barnes maze)	Bace1, Bdnf, Gsk3b	CpGs within promoter region of *Bace1* and *Gsk3b* analyzed CpGs in region upstream of *Bdnf* exon 4 analyzed	Pyrosequencing	↓ methylation of CpGs at TSS-554 and TSS-506 of *Bace1* promoter in hippocampus, of TSS-506 and TSS-518 in PFC, and of TSS-506 and TSS-554 in amygdala, and ↑ methylation of TSS-109 of *Bdnf* exon 4 region all associated with ↓ memory performance; no correlation with CpG sites in *Gsk3b*
	Makhathini et al. 2017^49^	Repetitive stress	Recognition memory (NOR)	–	Genome-wide	5-mC DNA Methylation ELISA	↓ global DNA methylation hippocampus in stressed group associated with impairment in memory recall
	Zhu et al. 2017^50^	Maternal separation	Memory (CFC), spatial learning and memory (MWZ)	Nr3c1^*^	17 CpG sites within promoter region of exon 1_7_	PCR and sequencing	↑ methylation of 14 out of 17 CpG sites within promoter region of exon 1_7_ in hippocampus and induced memory impairment after exposure to anesthesia

^s^self-report

*reported as GR in Zhu et al. 2017 study

Note: complete list of identified genes is not included for study Peter et al. 2016

↑ = increased; ↓ = decreased; CAARS Conors Adult Attention Rating Scale; CFC contextual fear conditioning; DLPFC dorsolateral prefrontal cortex; DML differentially methylated loci; FAB frontal assessment battery; MIS Buschke memory impairment screen; MMSE

mini-mental state examination; MWM Morris water maze; NA non-applicable; NOR novel object recognition; PFC prefrontal cortex; RM recognition memory; WASI Wechsler Abbreviated Scale of Intelligence

For human studies, bias was assessed using the validated Newcastle-Ottawa Scale, which evaluates the quality of non-randomized studies [31, 32]. A modification of the case-control scoring form, adopted from a previous systematic review [33], was used for the evaluation of three cross-sectional studies. Cross-sectional studies that received a score of six stars were considered to have low risk of bias; five or four stars were of medium risk of bias; and three stars or less were of high risk of bias. For four studies that had both case-control and cross-sectional designs, the scoring forms were combined into one form. This adjustment took the original case-control form and added the category “ascertainment of outcome.” Non-randomized studies with a score of ten were of low risk of bias; eight or nine were of medium risk of bias; and seven or less were of high risk of bias. One study [26] was excluded from bias assessment because the design was a randomized controlled trial but only the baseline characteristics of participants were used for analysis. Although prospective studies would have been the best study design to assess the temporal influence of stress on DNA methylation and subsequent outcomes of interest, none qualified to be in this review.

For animal studies, bias was evaluated using the SYRCLE’s risk of bias tool, with the use of signaling questions to facilitate the assessment [34]. Analysis was based on ten entries that relate to selection, performance, detection, attrition, reporting, and other biases. Within-table markings and bias scores for each risk item category were adopted from a previous systematic review [34, 35].

## Results

### Summary of included studies

Of the 16 full-text records included for narrative review, human and animal data were each extracted from 8 disparate studies. Of the eight human studies, four had CI as an outcome and four had subclinical CVD. Both male and female participants were represented in all but two studies, which had only male participants [36, 37]. Aside from one study [38] in which participants were, on average, seven years old when the study was conducted, average age was 43–69 years old. For studies with participants older than 50 years of age, age of stress exposure (including illness onset) was less than 50 [26, 36, 37, 39] (Additional files 5-6: Tables S4-S5). All except one human study [36] were judged to be of medium or high quality (Additional files 8-9: Tables S8-S9).

Of the eight animal studies, three had CI and five had subclinical CVD as an outcome. All studies were done with either mice or rats. All except one study [40], which had insufficient data, had the stressor applied before eight months of age (Additional file 7: Tables S6-S7). Overall risk of bias among studies was fairly high, with the greatest risk for allocation concealment and blinding. Low risk of bias was consistently reported for the following items: baseline characteristics, selective outcome reporting, and other bias. Risk items with unclear bias were random housing and random outcome assessment (Additional files 10-11: Tables S10-S11).

### Subclinical cardiovascular disease

#### Experimental groups

Data are presented with the manifestation of subclinical CVD listed first, followed by the measure taken to assess each respective marker (Table 1). In human studies, subclinical CVD was characterized by endothelial dysfunction [37, 38], atherosclerosis [26], and hypertension [39]. All markers were objectively assessed, except for in one study where hypertension was self-reported [39]. In animal studies, subclinical CVD was characterized by pulmonary hypertension [41, 42], hypertension [40], cardio-respiratory function [43], and endothelial dysfunction [44] (Table 1).

#### Stress exposure or illness diagnosis

In human studies, obstructive sleep apnea was evaluated in two studies [38, 39], stage 2–4 chronic kidney disease in one study [26], and war as a stressful life experience in another [37]. In animal studies, four evaluated hypoxia as a stress exposure [40, 41, 43, 44] and extrauterine growth restriction in another [42]. Presence or absence of control groups, as well as risk factors controlled for, are shown in Table S4, Additional file 5.

#### Genes studied and correlation with methylation

Among human studies, one [37] analyzed the exon 1_F_ promoter region of *NR3C1* in peripheral blood leukocytes and found a significant positive correlation between methylation of 12 CpG residues and brachial artery flow-mediated dilation. Analysis of self-reported hypertension in another study revealed an association with 636 differentially methylated loci across 22 genes [39]. Genes with greatest association are shown in Table 1. In a study by Kheirandish-Gozal et al. [38], greater methylation at one CpG site in the proximal promoter region of *NOS3* was associated with abnormal post-occlusive hyperemic responses. No correlation between global DNA methylation measured in blood leukocytes and either atherosclerosis or endothelial dysfunction was found among subjects with stage 2–4 chronic kidney disease in another study [26].

**Fig. 2:**
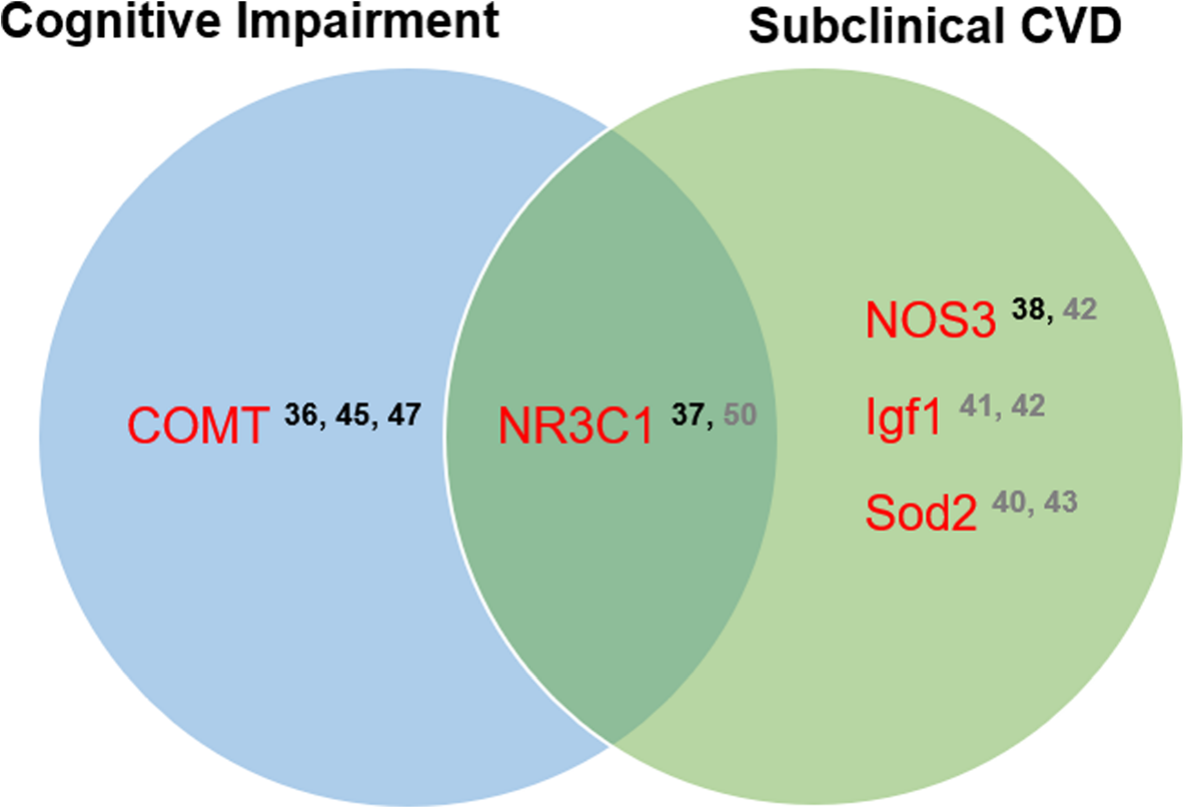
Genes identified in more than one study in association with cognitive impairment and/or subclinical CVD. Grey numbers = animal studies; black numbers = human studies; CVD = cardiovascular disease; COMT = catechol-O-methyltransferase; Igfl1 = insulin-like growth factor like family member 1; NOS3 = nitric oxide synthase 3; NR3C1 = nuclear receptor subfamily 3 group C member 1; Sod2 = superoxide dismutase 2. Numbered studies can be found in the References section

One animal study evaluated *Nos3*, but unlike results from a human study previously mentioned [38], there was no difference in total methylation at the promoter region in association with development of pulmonary hypertension [42]. However, this study found a positive association between hypertension and methylation at a CpG site in the promoter region of *Igfl1* and at several sites in *Fgfr2*, *Rhoc*, and *Notch1*, but not in *Med1.* A different CpG site in the promoter region of *Igfl1* showed a similar trend in a different study, with methylation positively correlated with pulmonary hypertension [41]. Among two studies evaluating *Sod2,* increased methylation of a single CpG site in the first intron (+ 128 from TSS) of *Sod2* was associated with increased cardio-respiratory dysfunction and hypertension when rats were exposed to intermittent hypoxia (IH) neonatally [43], and methylation of a single dinucleotide in CpG region 4, + 157 bp from the transcription start site (TSS) was positively associated with hypertension when rats were exposed to IH as adults [40]. In the latter study, increased hypertension was also associated with increased methylation of *Sod1*, *Txnrd2,* and *Prdx4*. Other genes evaluated include *Ace* and *Agt*, with a trend towards lower methylation of a CpG island in the *Ace* TSS and upstream of the *Agt* TSS, in association with greater endothelial dysfunction [44] (Table 1). Figure 2 illustrates the overlap of genes among studies with subclinical CVD as an outcome.

### Cognitive impairment

#### Experimental groups

Attention was evaluated by two human studies [45, 46], intelligence by one [45], memory by three [36, 46, 47], and learning, motor, global executive function, and cognitive mental state all together by two studies [36, 46]. All three animal studies [48–50] evaluated memory, including both recognition and spatial memory (Table 2).

### Stress exposure or illness diagnosis

In human studies, two conditions analyzed as stressors were schizophrenia and Human Immunodeficiency Virus (HIV) infection [36, 46]. Stressful experiences included postnatal malnourishment [45], and obstetric complications and/or stressful life events (self-reported) [47]. In animal studies, stressors included chronic variable stress [48], repetitive stress [49], and maternal separation [50]. Presence or absence of control groups, as well as risk factors controlled for, are shown in Additional file 6: Table S5.

### Genes studied and correlation with methylation

In one of the three human studies analyzing *COMT*, methylation in the promoter region was increased and moderately associated with ADHD index (measure of attention) in adults who were malnourished during the first year of life [45]. In another study there was significantly reduced methylation at the site of the Val158 allele of the rs4680 single nucleotide polymorphism (Val158Met) in association with greater impairment in working memory performance [47]. The Val158 allele creates a CpG site, whereas the alternate allele (Met) does not. A different study analyzing methylation of *MB-COMT,* which is the long form isoform of *COMT*, found no association between adult-onset schizophrenia, CI, and methylated CpGs in the dorsolateral prefrontal cortex (DLPFC) [36]. This study was considered to be of high risk of bias, with sample size being a large issue of concern (Additional file 9: Table S9). With only 3 DLPFC control samples to compare to an experimental sample size of 15, it is unlikely that any effect could be observed. In the same study, *DISC1, DRD1, GABBR2, HINT1, RELN,* and *SLC6A4* were analyzed in the hippocampus, cerebellum, or anterior cingulate cortex, but no significant associations were observed. *SLC6A4* codes for the serotonin receptor and methylation changes in this gene have been implicated in mice socially isolated as adults [51]. Other genes analyzed by studies were *ZBTB9* and *SYNGAP1,* with significantly increased methylation related to higher intelligence [45], but after adjusting for socioeconomic status, significance in CpG sites in the island and shore regions of *ZNF57* was lost. An additional 15 X-linked differentially methylated loci in males and one in females were associated with both attention and intelligence. Details on these X-linked loci, as well as on a myriad of additional genes with significant methylation changes, including *ABCF1, IFNG, MIR200B, VARS,* and *WT1,* can be found in the supplemental files of Peter et al. [45]. In another study, HIV-associated neurocognitive disorders (HAND) was correlated with increased methylation of 363 CpGs genome-wide in the occipital cortex [46]. However, this was for overall cognitive function and not for individual cognitive domains (learning, memory, attention, motor) (Table 2).

Among animal studies, four genes were analyzed for methylation changes. In animals who were physically, psychologically, and socially stressed, reduced methylation of CpGs located at tss-554 and tss-506 of the *Bace1* promoter region in the left side of the hippocampus was negatively correlated with memory performance [48]. In the prefrontal cortex, reduced methylation was examined at tss-506 and tss-518, but the latter occurred only among aged stressed mice (vs. young stressed mice). Lower methylation was observed at tss-506 and tss-554 in the amygdala, with the latter again only seen among aged stressed mice. In the same study, tss-109 of *Bdnf* exon 4 region had increased methylation in the hippocampus of aged mice, and no effects were observed for *Gsk3b* [48]. In a study by Zhu et al. [50], 14 out of 17 CpG sites within the promoter region of exon 1_7_ of *Nr3c1* had increased methylation in the hippocampus of rats with neonatal maternal separation, which was associated with memory impairment [50]. In comparison, global methylation in the hippocampus was negatively correlated with recognition memory in another study where rats were repetitively stressed; however, no specific genes were further identified [49] (Table 2). These results are consistent with previous research showing that regulation of learning, memory, and basic brain function is associated with methylation of stress response genes in the hippocampus, and stressors like childhood abuse and social environment can modify this methylation [19, 52]. Figure 2 illustrates the overlap of genes among studies with CI as an outcome.

## Discussion

To the best of our knowledge this is the first review to examine the effects of stress on DNA methylation associated with subclinical CVD and CI. Existing reviews have analyzed differentially methylated genes in either outcome of interest, but not with the stress component discussed in this review [15]. For reviews that have evaluated some stress exposure, either only one outcome of interest was examined, or genes were indirectly associated with one of the outcomes [19, 52, 53]. Therefore, we conducted a review to highlight what information already exists and what gaps still remain in the literature (Fig. 1). DNA methylation can be a powerful biomarker for early detection of onset and progression of CVD and CI.

The biggest difficulty with this review was in determining what would classify as a stressful exposure. The experience of stress can be subjective, with the same incident being stressful for one person and not at all stressful for another. Psychological and physiological stress can be considered as distinct concepts, so we kept our inclusion of stress as broad as possible. Independent of stress, life events like illness would indisputably have some direct biological effects on methylation and progression of CVD or CI, but this doesn’t mean that the effects of stress would be negligible. Inclusion of animal studies with hypoxia as a stressor may be comparable to obstructive sleep apnea in humans, and methylation changes specific to stress may be parsed out from those that underly the biological effects of the illness or other factors.

For studies aiming to identify the moderating effects of stress on DNA methylation, it is advisable that a subjective survey be used to quantify study participants’ perception of stress. Studies can better gauge life stress through use of interviews like the Stress and Adversity Inventory (STRAIN), due to its breadth of coverage. This assessment measures life course stress by covering various life domains and quantitatively calculates the number of stressors within each domain, from early-life throughout adulthood, and the self-reported severity of those stressors [54]. One study from this review [46] concluded that duration of HIV infection contributed to differential DNA methylation levels, with viral DNA/RNA levels not contributing to this effect since there were no observed differences between control and experimental groups. This could imply that stress duration associated with the illness influenced these results. However, the sample size for the neurocognitive control group was 8, and 18 for the HAND group. Larger sample sizes might have increased the power and thus revealed a significant difference in viral levels; therefore, experienced stress due to illness duration might not actually be the driving factor. With depression and stress explaining some variability in the disease progression of HIV [55], it is possible that independent stressful life events might have moderated some of the effects observed with DNA methylation. This might also have been the case in one study [36] in this review which analyzed brain samples of schizophrenic patients. Larger sample sizes, along with comprehensive stress survey results, might have captured differences in DNA methylation, due to the relation of stress with psychiatric illness [14]. It is highly recommended that future studies use adequate sample sizes, so to omit this as a limiting factor in the absence of any observed effects of stress.

With the brain being more sensitive to plasticity at certain points throughout development, it is important to consider the temporal nature of stress [56]. A stressed individual during a particular developmental period may be more vulnerable to enduring epigenetic alterations that could predispose them to disease onset. Recent research with participants from the Multi-Ethnic Study of Atherosclerosis (MESA), after accounting for socioeconomic status (SES), found that living in a disadvantaged neighborhood during early-life was associated with increased DNA methylation of stress- and inflammation-related genes assessed later in life [57]. It is possible that additional changes might have been observed had more stressful experiences been assessed, with cumulative exposure previously reported to affect methylation [52]. Previous research has shown that the occurrence of three or more childhood adversities was associated with adult-onset of physical conditions such as heart disease and T2DM [58]. One study in our review utilized stressful life events questionnaires with a life-history calendar in conjunction with self-reported obstetric complications, and scores were differentiated into lower or greater stress [47]. Greater stress was associated with lower methylation in *COMT* and greater CI than with lower stress. However, there was no prospective study design which measured methylation before onset of any CI or subclinical CVD, so it cannot be adequately concluded whether DNA methylation is a predisposing factor for the outcomes reported or a consequence of them.

Incongruent results among studies could also be attributed to the effects of other risk factors on methylation. For CVD these include blood pressure, smoking, nutrition, pollution, cholesterol, and diabetes [59–64]. In this review, blood pressure was only accounted for in two studies [37, 38], smoking in three [26, 37, 39], cholesterol in two [38, 39], and diabetes in two [37, 39]. Information on risk factors could not be determined for two studies [36, 47]. Anxiety and depression are possible modifying factors when assessing cognitive impairment, due to the potential effects these affective disorders have on confounding cognitive testing performance [65]. One study controlled for depressive symptoms, but cognitive performance was not an outcome of interest [37].

Using traditional risk factors to predict CVD proves more difficult in explaining variability in outcomes for women than for men. Empirical evidence for coronary heart disease risk suggests that men have a greater lifetime risk than women for development of disease [66], but higher rates of all-cause mortality exist for women, at any level of coronary calcification, even after adjustment for traditional risk factors [67]. When considering both sex and risk factors in a population of diabetic subjects, men had a 2.1 risk and women a 4.9 risk for cardiovascular death, compared to non-diabetic subjects [68]. CVD typically occurs later in life for women, and there may be some sex-specific epigenetic marks, as reported previously [61]. All but two human studies [36, 37] in this review included both sexes in their study design, with one [45] finding X-linked differentially methylated loci. Only one study [50] in this report included animals of each sex (Additional file 7: Tables S6-S7). Future studies should consider recruitment of both sexes.

Ethnic and racial disparities also contribute to differences in CVD and CI risk. In a comparison of European Americans and African Americans (AA) in the Diabetes Heart Study [2], AA had increased carotid artery intima-medial thickness, as well as risk factors like smoking and low-density lipoprotein cholesterol. However, they had lower levels of coronary artery calcium and carotid calcified plaque. This differential pathogenesis might be partly due to epigenetic variations that include DNA methylation. For example, it has been shown that baseline endothelial cell gene expression in healthy young adults is differentially expressed in 31 genes between AA and European Americans [69]. This could contribute to the disparity observed in vascular-related disease among AA. In one study using participants from MESA [70], CVD risk factor ankle-brachial index (ABI) was analyzed in four different ethnic groups [71]. Controlling for traditional and novel risk factors, AA had a 1.47 greater risk for peripheral arterial disease (PAD) compared with non-Hispanic whites, followed by Hispanics and Chinese. When normal ABI levels were analyzed in regards to gender and ethnicity in a subgroup of MESA participants, women had lower ABI values than men, and blacks had lower levels than non-Hispanic whites [72]. One study [46] in this review had participants of two different ethnicities, but no sensitivity analysis was done to identify potential differences in methylation. These results further highlight the need for future studies to control for sex, race, and ethnicity, in addition to assessing differences across strata, such as that of stress on methylated genes in men and women of different ethnic groups.

Variability in methylation changes of the same gene could be attributed to the method of methylation assessment used and which cells or tissues were analyzed. For example, the study by Peter et al. [45] used the Infinium HumanMethylation450 Bead Chip Array, which detects methylation status of over 485,000 individual CpGs in 99% of known genes, including coding regions and island shores [73]. With this method, increased methylation was identified at two sites (cg06860277, cg07194846) within the promoter region of *COMT*, in addition to sites in shore and sea regions of various genes [74]. These sites might have been overlooked had the methylation assessment been pyrosequencing, for example, which requires initial knowledge of individual target CpG site sequences. Alelu-Paz et al. [36] reported no methylation change in *MB-COMT*, but analysis was restricted to brain regions. Significant methylation changes in *COMT* were found in studies which utilized peripheral blood mononuclear cells and whole blood [45, 47]. Results from whole blood analyses should be interpreted with caution, due to reported differences in methylation between white blood cell fractions [75]. Only one study in this review controlled for methylation across different cell fractions [45]. Moreover, Ursini et al. [47] showed that methylation at the rs4680 site and at sites within the promoter region of *COMT* was dependent on genotype; individuals with two Val alleles had lower methylation at all sites and greater working memory impairment. These sites are likely different than those reported to have increased methylation in the study by *Peter et. al* [45]. For future studies aiming to find novel genes and specific CpG sites, the best approach would be to search for differentially methylated regions across the genome, with consideration of well-established stress-related risk genotypes like that for *FKBP5, NR3C1,* and *COMT* [76].

Our findings supplement work done with epigenetic regulation of genes associated with stress, subclinical CVD, and cognition. With no single study analyzing common genes between subclinical CVD and cognition, and the high comorbid prevalence of the two, future work should aim to combine these areas (Fig. 1). There was one common gene of interest (*NR3C1*) which was associated with stress and both outcomes, albeit not in the same animal or study [37, 50]. Nonetheless, there lies the implication that other genes with similar association can be identified with methodologically rigorous studies. For example, although *COMT* methylation was only analyzed in association with CI, it is likely that studies assessing subclinical CVD may also find a relationship. One study [40] in this review found significantly increased methylation at several CpG sites in the gene *Txndr,* which lies adjacent to *COMT* in both humans and rats. Therefore, some of these CpG sites of significance might overlap if both genes were analyzed. Since common genes have been reported in both CVD and pathological disease processes like Alzheimer’s Disease [22], future studies with elderly participants may consider controlling for these processes if they wish to explore CI as an outcome that is independent of these processes.

Despite limitations, this review provides background for future population-based studies designed to identify DNA methylation patterns of overlapping genes between subclinical CVD and CI. These patterns may be critical to identifying links between stress and those vulnerable to future health disparities. We limited our literature search to Pubmed, which may have affected the number of included studies. Additionally, no comparison of statistical approaches was performed, largely because some studies lacked complete methodological information. It was beyond the scope of this review to evaluate the statistical approaches for each study, but we understand the importance of this and had there been less variability across studies, we would have addressed this. The exclusion of prenatal and parental stress may have left out pertinent data, but those data are beyond the scope of this review, which was to analyze stress exposed from early- life (neonatal) to mid-life.

## Conclusions

Measuring life course stress with validated scales, accounting for number of stressors, utilizing genome-wide methylation analysis, controlling for sex, ethnicity, and traditional and novel risk factors, are all important considerations for future studies. Early detection of vulnerable individuals with biological biomarkers can pave the way for clinical intervention programs to reduce the burden of CVD and comorbidities highly ubiquitous today.

## Additional files

Additional file 1: **Table S1.** PRISMA 2009 Checklist. (DOCX 24 kb)

Additional file 2: Search strategy. (DOCX 12 kb)

Additional file 3: **Tables S2.** and **Table S3.**. Number of excluded studies after title and abstract screening, and after full-text screening. (DOCX 20 kb)

Additional file 4: **Figure S1.** Flow diagram of study selection. (DOCX 129 kb)

Additional file 5: **Table S4.**. General characteristics of human studies investigating subclinical cardiovascular disease. (DOCX 21 kb)

Additional file 6: **Table S5.** General characteristics of human studies investigating cognitive impairment. (DOCX 20 kb)

Additional file 7: **Tables S6** and **Table S7.** General characteristics of animal studies investigating association of DNA methylation with stress and cognitive impairment, and stress and subclinical cardiovascular disease, respectively. (DOCX 22 kb)

Additional file 8: **Table S8.** Quality ratings for the three cross-sectional studies included. (DOCX 27 kb)

Additional file 9: **Table S9.** uality ratings for the four case-control/cross-sectional studies included. (DOCX 39 kb)

Additional file 10: **Table S10.** Risk of bias for animal studies. (DOCX 16 kb)

Additional file 11: **Table S11.** Risk of bias score for each risk item in animal studies. (DOCX 31 kb)

## Abbreviations

AA: African Americans
ABI: Ankle-brachial index
CI: Cognitive impairment
CVD: Cardiovascular disease
HAND: HIV-associated neurocognitive disorders
HIV: Human Immunodeficiency Virus
IH: Intermittent hypoxia
MESA: Multi-Ethnic Study of Atherosclerosis
SES: Socioeconomic status
STRAIN: Stress and Adversity Inventory
TSS: Transcription start site
