# Supplementary material for: Effects of early- and mid-life stress on DNA methylation of genes associated with subclinical cardiovascular disease and cognitive impairment: a systematic review
Source: BMC Med Genet. 2019 Mar 12;20:39. doi: 10.1186/s12881-019-0764-4 (PMC6417232; doi:10.1186/s12881-019-0764-4)
Supplement: Supplementary file 2 — Search strategy. (DOCX 12 kb) [file 12881_2019_764_MOESM2_ESM.docx]

((((Atherosclerotic plaques[tiab] OR Atherosclerotic plaque[tiab] OR Carotid artery calcium[tiab] OR Carotid artery calcifications[tiab] OR Carotid artery calcification[tiab] OR Coronary artery calcium[tiab] OR Coronary artery calcifications[tiab] OR Coronary artery calcification[tiab] OR Subclinical CVD[tiab] OR Subclinical cardiovascular disease[tiab] OR Subclinical atherosclerosis[tiab] OR Subclinical arteriosclerosis[tiab] OR Intima media thickness[tiab] OR Left ventricular mass[tiab] OR Biventricular volume[tiab] OR Calcified plaque[tiab] OR High blood pressures[tiab] OR High blood pressure[tiab] OR Hypertension[tiab] OR Carotid intima-media thickness[MeSH] OR Hypertension[MesH:noexp] OR Plaque, atherosclerotic[MeSH] OR ventricular function, left[MesH] OR left ventricular function[tiab] OR ankle brachial index[Mesh] OR ankle brachial index[tiab] OR ankle brachial indices[tiab] OR atherogenesis[tiab])) OR (Cognition[MeSH:NoExp] OR Cognition Disorders[Mesh:NoExp] OR Cognitive Dysfunction[Mesh] OR Neuropsychological tests[MesH] OR Executive function[MesH] OR Neuropsychological tests[tiab] OR neuropsychological testing[tiab] OR Cognitive performance[tiab] OR Cognitive Function[tiab] OR Cognitive dysfunction[tiab] OR Cognitive dysfunctions[tiab] OR Cognitive deficit[tiab] OR Cognitive deficits[tiab] OR Cognitive impairment[tiab] OR Cognitive impairments[tiab] OR Neurocognitive disorder[tiab] OR Neurocognitive disorders[tiab] OR Cognitive decline[tiab] OR Cognitive declines[tiab] OR executive function[tiab]))) AND (Epigenomics[MeSH] OR Cpg islands[MeSH] OR DNA methylation[MeSH] OR Epigenomics[tiab] OR Epigenetics[tiab] OR DNA methylation[tiab] OR DNA methylations[tiab] OR DNA hypermethylation[tiab] OR DNA hypomethylation[tiab] OR differentially methylated regions[tiab] OR hydroxymethylation[tiab]OR cpg[tiab] OR ((cluster[tiab] OR clusters[tiab] OR island[tiab] OR islands[tiab] OR site[tiab] OR sites[tiab]) AND (cpg-rich[tiab] OR non-cpg[tiab]))).

**Search Terms.** A complete list of the combination of methylation terms AND either cognitive impairment OR subclinical cardiovascular disease terms. Subclinical CVD terms are common clinical markers for CVD and were abstracted from review papers, and papers within them. CI terms were those that related to cognition, without regard for any specific condition. All terms were refined using MeSH categories in the Pubmed database.
