# Supplementary material for: Effects of early- and mid-life stress on DNA methylation of genes associated with subclinical cardiovascular disease and cognitive impairment: a systematic review
Source: BMC Med Genet. 2019 Mar 12;20:39. doi: 10.1186/s12881-019-0764-4 (PMC6417232; doi:10.1186/s12881-019-0764-4)
Supplement: Supplementary file 3 — Tables S2. and Table S3.. Number of excluded studies after title and abstract screening, and after full-text screening. (DOCX 20 kb) [file 12881_2019_764_MOESM3_ESM.docx]

**Table S2.** Number of excluded studies after title and abstract screening.

| EXCLUSION CRITERIA | # STUDIES |
| --- | --- |
| No direct stress exposure in early- or mid-life | 527 |
| Paternal/prenatal stress exposure, NOT neo/postnatal, as direct cause | 106 |
| Subclinical CVD/cognitive impairment is not directly measured | 79 |
| Cognitive impairment/subclinical CVD is major symptom of illness (e.g. Alzheimer’s) | 52 |
| No clear direct association between stress, subclinical CVD or cognitive impairment, and DNA methylation | 41 |
| No DNA methylation | 37 |
| Stress event is environmental chemical exposure | 9 |
| Study not yet completed | 1 |

**Table S3.** Number of excluded studies after full-text screening.

| EXCLUSION CRITERIA | # STUDIES |
| --- | --- |
| No direct stress exposure in early- or mid-life | 12 |
| Paternal/prenatal stress exposure, NOT neo/postnatal, as direct cause | 2 |
| Subclinical CVD/cognitive impairment is not directly measured | 10 |
| No clear direct association between stress, subclinical CVD or cognitive impairment, and DNA methylation | 9 |
| No DNA methylation | 1 |
| No full-text available | 1 |
