# Supplementary figures and images for: Effects of early- and mid-life stress on DNA methylation of genes associated with subclinical cardiovascular disease and cognitive impairment: a systematic review
Source: BMC Med Genet. 2019 Mar 12;20:39. doi: 10.1186/s12881-019-0764-4 (PMC6417232; doi:10.1186/s12881-019-0764-4)

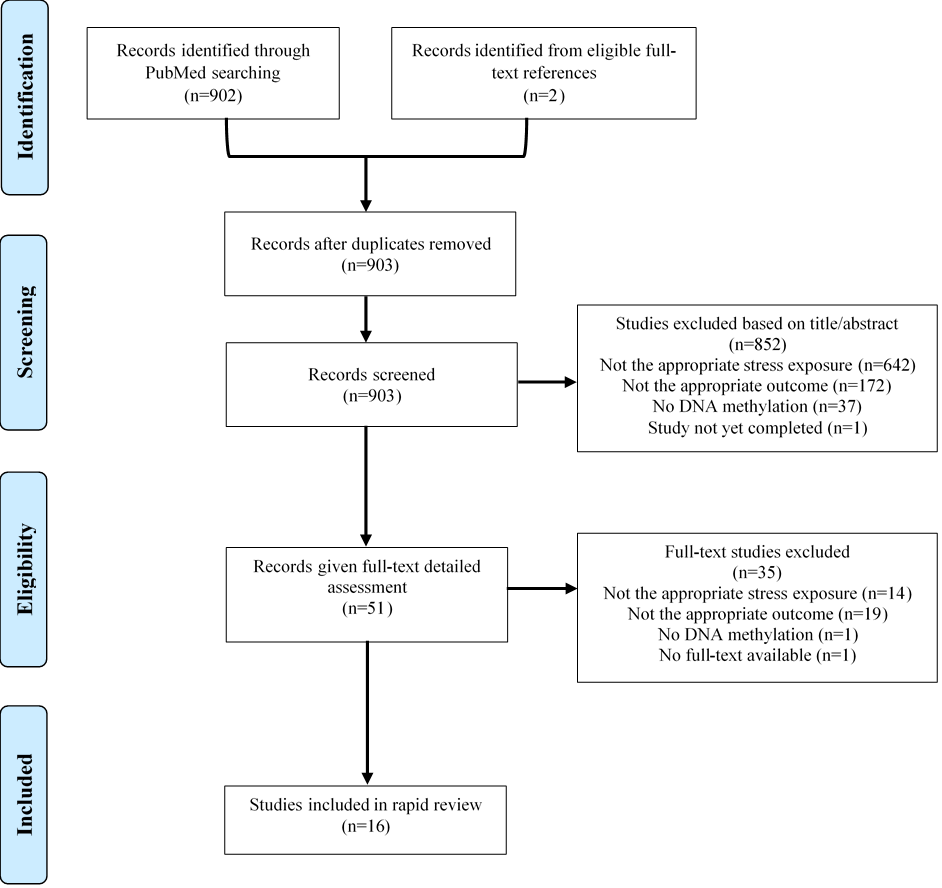


**Figure S1.** Flow diagram of study selection.

Supplement: Supplementary file 4 — Figure S1. Flow diagram of study selection. (DOCX 129 kb) [file 12881_2019_764_MOESM4_ESM.docx]

**Table S11.** Risk of bias score for each risk item in animal studies.


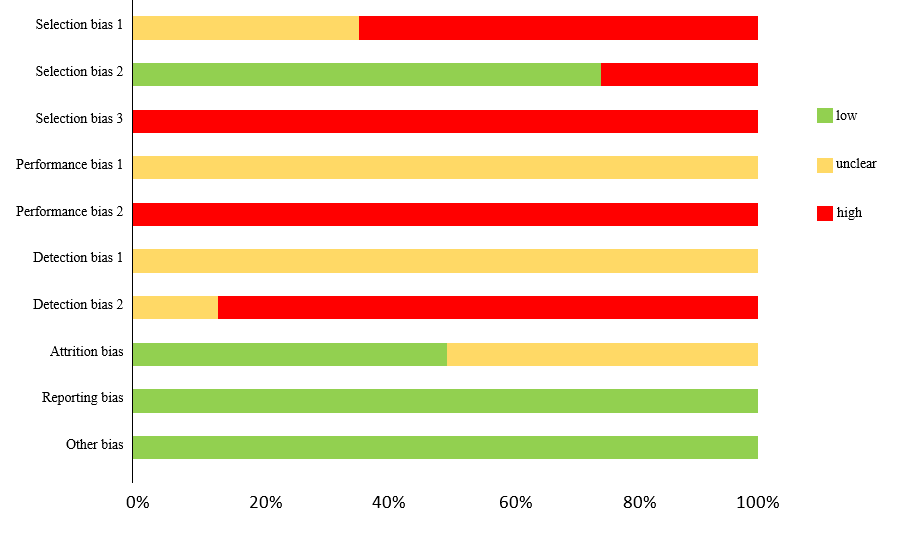

Supplement: Supplementary file 11 — Table S11. Risk of bias score for each risk item in animal studies. (DOCX 31 kb) [file 12881_2019_764_MOESM11_ESM.docx]
