# Supplementary material for: Effects of early- and mid-life stress on DNA methylation of genes associated with subclinical cardiovascular disease and cognitive impairment: a systematic review
Source: BMC Med Genet. 2019 Mar 12;20:39. doi: 10.1186/s12881-019-0764-4 (PMC6417232; doi:10.1186/s12881-019-0764-4)
Supplement: Supplementary file 5 — Table S4.. General characteristics of human studies investigating subclinical cardiovascular disease. (DOCX 21 kb) [file 12881_2019_764_MOESM5_ESM.docx]

**Table S4.** General characteristics of human studies investigating subclinical cardiovascular disease.

| **Lead Author, Publication Date** | **Study Design** | **Location of Study** | **Sample size/characterization** | **Gender**  **(males %)** | **Age**  **(mean years + SD)** | | **Risk Factors Adjusted For** | **Outcome(s)** | **Quality** |
| --- | --- | --- | --- | --- | --- | --- | --- | --- | --- |
| Chen et al. 2016^39^ | Case-control  Cross-sectional | Taiwan | *Discovery cohort*  healthy subjects: 6  OSA subjects: 15 | 5(83.3)  13(86.7) | 51.0+14.2  51.8+8.9 | Alcoholism (%), API, BMI, CKD, cholesterol, COPD/asthma, diabetes mellitus, heart disease, ODI, smoking (%), stroke, triglycerides | | Disease severity, EDS, HTN | 9/10 |
|  |  |  | *Validation cohort*  primary snoring subjects: 24  OSA subjects: 48 | 19(79.2)  40(83.3) | 47.8 + 12.2  48.8 + 12.2^a^ |  |  |  |  |
| Kheirandish-Gozal et al. 2013^38^ | Case-control  Cross-sectional | USA | Controls: 35  Obstructive sleep apnea: 36 | 25(71)  22(61) | 7.4 + 1.8  With endothelial dysfunction (7.3 + 2.2); without endothelial dysfunction (7.5 + 2.5) | AHI, blood pressure, BMI, total cholesterol, HDL, LDL, triglycerides | | Endothelial dysfunction | 9/10 |
| Nanayakkara et al. 2008^26^ | NA | The Netherlands | Stage 2-4 CKD subjects: 78 | 43(55) | 53 + 10 | BMI, C-reactive protein smoking, use of ACE inhibitors, plasma homocysteine, renal function, smoking (%) | | Atherosclerosis, endothelial dysfunction | NA |
| Zhao et al. 2015^37^ | Cross-sectional | USA | Vietnam war veterans: 168 (84 pairs of monozygotic twins) | (100) | 55.1 + 2.8 | Alcohol consumption, blood pressure, BMI, depressive symptoms, fasting glucose, hypertension, inflammatory biomarkers, lipids, physical activity, PTSD symptoms, smoking, T2D | | Atherosclerosis | 5/6 |

AA= African Americans; AHI= apnea hypopnea index; ATIC= Anti-Oxidant Therapy in Chronic Renal Insufficiency; BMI= body mass index; EA= European Americans; CKD= chronic kidney disease; COPD= chronic obstructive pulmonary disease; CVD=cardiovascular disease; DC= discovery cohort; EDS= excessive daytime sleepiness; F=female; HTN= hypertension; M=male; NA= non-applicable; ODI= oxygen desaturation index; SES= socioeconomic status; T2D= type 2 diabetes; VC= validation cohort
