# Supplementary material for: Effects of early- and mid-life stress on DNA methylation of genes associated with subclinical cardiovascular disease and cognitive impairment: a systematic review
Source: BMC Med Genet. 2019 Mar 12;20:39. doi: 10.1186/s12881-019-0764-4 (PMC6417232; doi:10.1186/s12881-019-0764-4)
Supplement: Supplementary file 6 — Table S5. General characteristics of human studies investigating cognitive impairment. (DOCX 20 kb) [file 12881_2019_764_MOESM6_ESM.docx]

**Table S5.** General characteristics of human studies investigating cognitive impairment.

| **Lead Author, Publication Date** | **Study Design** | **Location of study** | **Sample size/characterization** | **Gender**  **(male %)** | **Age**  **(mean years + SD)** | **Risk Factors Adjusted For** | **Outcome** | **Quality** |
| --- | --- | --- | --- | --- | --- | --- | --- | --- |
| Alelu-Paz et al. 2015^36^ | Case-control  Cross-sectional | Spain | Total: 33 | (100) | 68.8 + 14.4  77.6 + 10.1 | NA | Cognitive status | 8/10 |
|  |  |  | Controls: 4  Schizophrenic subjects: 29 |  |  |  |  |  |
| Levine et al. 2017^46^ | Cross-sectional | USA | HIV subjects: 58 | 46(79) | 46.5 + 1.9  45.7 + 9 | Cellular composition, CD4+ cell count, CPE, education, HIV DNA & RNA, illness duration*, viral load | HAND diagnosis (n=43) | 6/6 |
| Peter et al. 2016^45^ | Case-control  Cross-sectional | USA | Total: 168 | 24(54.6)  23(46.0) | 43.9 + 2.0  44.6 + 1.8 | Childhood SES*, childhood IQ, white blood cell composition | Cognition, attention | 9/10 |
|  |  |  | Controls: 44  Postnatally malnourished subjects: 50 |  |  |  |  |  |
| Ursini et al. 2011^47^ | Cross-sectional | Italy | Healthy subjects: 84 | 32(38.1) | 25.9 + 5.6 | NA | Working memory | 4/6 |

* significant difference between groups

AA= African Americans; EA= European Americans; CNTN= California NeuroAIDS Tissue Network; CVD=cardiovascular disease; CPE= CNS penetration effectiveness; F=female; HAND=HIV-associated neurocognitive disorders; M=male; NA= non-applicable; NNAB: National Neurological AIDS Bank; SES= socioeconomic status; T2D= type 2 diabetes
