# Supplementary material for: Effects of early- and mid-life stress on DNA methylation of genes associated with subclinical cardiovascular disease and cognitive impairment: a systematic review
Source: BMC Med Genet. 2019 Mar 12;20:39. doi: 10.1186/s12881-019-0764-4 (PMC6417232; doi:10.1186/s12881-019-0764-4)
Supplement: Supplementary file 7 — Tables S6 and Table S7. General characteristics of animal studies investigating association of DNA methylation with stress and cognitive impairment, and stress and subclinical cardiovascular disease, respectively. (DOCX 22 kb) [file 12881_2019_764_MOESM7_ESM.docx]

**Table S6.** General characteristics of animal studies investigating association of DNA methylation with stress and cognitive impairment.

| **Lead Author, Publication Date** | **Experimental Group** | **Sample Size** | **Sex**  **(males %)** | **Age at Outcome Assessment** | **Outcome(s)** |
| --- | --- | --- | --- | --- | --- |
| Cordner et al. 2016^48^ | Recurrent physical, psychological, and social stress for 14 days at 6 or 8 months old | 5-6 mice per group | (100) | 6.5-8.5 months old | Recognition memory, spatial learning and memory |
| Makhathini et al. 2017^49^ | Repetitive restraining for 6h for 28 consecutive days at P70 | 9 rats per group | (100) | P92-P95 | Recognition memory |
| Zhu et al. 2017^50^ | Maternal separation from P1-P21 and sevoflurane inhalation at P85 | 10 rats per group | NA* | P86 | Memory, spatial learning and memory |

*number unknown but both sexes included

**Table S7.** General characteristics of animal studies investigating association of DNA methylation with stress and subclinical cardiovascular disease.

| **Lead Author, Publication Date** | **Experimental Group** | **Sample Size** | **Sex**  **(males %)** | **Age at Outcome Assessment** | **Outcome(s)** |
| --- | --- | --- | --- | --- | --- |
| Chu et al. 2015^44^ | Postnatal intermittent hypoxia for 6h per day for 4 weeks | 8 mice per group | (100) | 3 months | Endothelial dysfunction |
| Nanduri et al. 2012^43^ | Intermittent hypoxia for 8h per day for 10 days at P0-P10 | 10 rats per group | NA* | P40-P50 | Cardio-respiratory function |
| Nanduri et al. 2017^40^ | Long-term (30 days) intermittent hypoxia at adult age | 8 rats per group | (100) | After 30-day recovery period, following hypoxia exposure | Hypertension |
| Yang et al. 2015^41^ | Normobaric hypoxia for 2 weeks shortly after birth | NA** | NA** | P14 | Pulmonary hypertension |
| Zhang et al. 2014^64^ | Extrauterine growth restriction within 12h of birth | > 20 rats per group | (100) | P63 | Pulmonary hypertension |

*number unknown but both sexes included; **number of mice used per group and sex of them is unknown
