# Supplementary material for: Effects of early- and mid-life stress on DNA methylation of genes associated with subclinical cardiovascular disease and cognitive impairment: a systematic review
Source: BMC Med Genet. 2019 Mar 12;20:39. doi: 10.1186/s12881-019-0764-4 (PMC6417232; doi:10.1186/s12881-019-0764-4)
Supplement: Supplementary file 8 — Table S8. Quality ratings for the three cross-sectional studies included. (DOCX 27 kb) [file 12881_2019_764_MOESM8_ESM.docx]

| **Lead Author, Publication Date** | **Selection**  (Max 1 star) | | | | **Comparability**  (score) | | **Outcome**  (score) | | | **Total Score (# stars, Max=6)** | **Comments** |
| --- | --- | --- | --- | --- | --- | --- | --- | --- | --- | --- | --- |
|  | Representativeness of Exposed Group | Selection of Non-Exposed Group | Ascertainment of Exposure | Demonstration that Outcome Not Present at Study Start: Not Applicable | Comparability of exposed and non-exposed groups on the basis of the design or analysis (age, gender?) | | Ascertainment of outcome | Follow-Up Long Enough: Not Applicable | Adequacy of Follow-Up of Cohorts: Not Applicable |  |  |
| Levine et al. 2017^46^ |  |  |  | NA |  |  |  | NA | NA | 5 | When controlling for HIV RNA and DNA levels, control group sample size was very small in comparison to exposed group |
| Ursini et al. 2011^47^ |  |  |  | NA |  |  |  | NA | NA | 4 | No mention of where subjects were recruited from |
| Zhao et al. 2015^37^ |  |  |  | NA |  |  |  | NA | NA | 5 |  |

**Table S8.** Quality ratings for the three cross-sectional studies included.
