# Supplementary material for: Effects of early- and mid-life stress on DNA methylation of genes associated with subclinical cardiovascular disease and cognitive impairment: a systematic review
Source: BMC Med Genet. 2019 Mar 12;20:39. doi: 10.1186/s12881-019-0764-4 (PMC6417232; doi:10.1186/s12881-019-0764-4)
Supplement: Supplementary file 9 — Table S9. uality ratings for the four case-control/cross-sectional studies included. (DOCX 39 kb) [file 12881_2019_764_MOESM9_ESM.docx]

| **Lead Author, Publication Date** | **Selection**  (Max 1 star) | | | | **Comparability**  (Max 2 stars) | | **Exposure**  (Max 1 star) | | | **Outcome**  (Max 1 star) | **Total # Stars**  **(Max=10)** | **Comments** |
| --- | --- | --- | --- | --- | --- | --- | --- | --- | --- | --- | --- | --- |
|  | Case definition | Representative of cases | Selections of controls | Definition of controls | Comparability of cases and controls on the basis of the design or analysis (age, gender?) | | Ascertainment of exposure | Same method of ascertainment for participants | Non-response rate | Ascertainment of outcome |  |  |
| Alelu-Paz et al. 2015^36^ |  |  |  |  |  |  |  |  |  |  | 7 | No risk factors controlled for; control group sample size is very small in comparison to case group |
| Chen et al. 2016^39^ |  |  |  |  |  |  |  |  |  |  | 9 | Control sample size is low (1/2 of case subjects); # of non-respondents mentioned but not discussed |
| Kheirandish-Gozal et al. 2013^38^ |  |  |  |  |  |  |  |  |  |  | 9 |  |
| Peter et al. 2017^45^ |  |  |  |  |  |  |  |  |  |  | 9 | describes non-respondents for loss-of follow up but no mention of difference in rate for both groups |

**Table S9.** Quality ratings for the four case-control/cross-sectional studies included.
