# Supplementary material for: Effects of early- and mid-life stress on DNA methylation of genes associated with subclinical cardiovascular disease and cognitive impairment: a systematic review
Source: BMC Med Genet. 2019 Mar 12;20:39. doi: 10.1186/s12881-019-0764-4 (PMC6417232; doi:10.1186/s12881-019-0764-4)
Supplement: Supplementary file 10 — Table S10. Risk of bias for animal studies. (DOCX 16 kb) [file 12881_2019_764_MOESM10_ESM.docx]

|  | **1** | **2** | **3** | **4** | **5** | **6** | **7** | **8** | **9** | **10** |
| --- | --- | --- | --- | --- | --- | --- | --- | --- | --- | --- |
| **Lead Author, Publication Date** | **Selection bias** | | | **Performance bias** | | **Detection bias** | | **Attrition bias** | **Reporting bias** | **Other bias** |
|  | Sequence generation | Baseline characteristics | Allocation concealment | Random housing | Blinding | Random outcome assessment | Blinding | Incomplete outcome data | Selective outcome reporting | Other sources of bias |
| Chu et al. 2015^44^ | x | ✓ | x | ? | x | x | x | ✓ | ✓ | ✓ |
| Cordner et al. 2015^48^ | ? | ✓ | x | ? | x | x | ? | ? | ✓ | ✓ |
| Makhathini et al. 2017^49^ | x | ✓ | x | ? | x | x | x | ✓ | ✓ | ✓ |
| Nanduri et al. 2012^43^ | x | x | x | ? | x | x | x | ? | ✓ | ✓ |
| Nanduri et al. 2017^40^ | x | ✓ | x | ? | x | x | x | ? | ✓ | ✓ |
| Yang et al. 2015^41^ | x | x | x | ? | x | x | x | ? | ✓ | ✓ |
| Zhang et al. 2014^42^ | ? | ✓ | x | ? | x | x | x | ✓ | ✓ | ✓ |
| Zhu et al. 2017^50^ | ? | ✓ | x | ? | x | x | x | ✓ | ✓ | ✓ |
| 1. 1 ✓ = Adequate randomization; ? = randomized but no details; x = no evidence of randomization. 2. 2 ✓ = Baseline characteristics given; x = baseline characteristics not given. 3. 3 ✓ = Evidence of adequate concealment of groups; x = no evidence of adequate concealment of groups. 4. 4 ✓ = Evidence of random housing of animals; ?  = unknown housing arrangement. 5. 5 ✓ = Evidence of caregivers blinded to intervention; x = no evidence of caregivers blinded to intervention. 6. 6 ✓ = Evidence of random selection for assessment; x = no evidence of random selection for assessment. 7. 7 ✓ = Evidence of assessor blinded; ?= assessor blinded for only one main outcome; x = no evidence of assessor blinded. 8. 8 ✓ = Explanation of missing animal data; ? = initial sample size of animals unknown; x = no explanation of missing animal data. 9. 9 ✓ = Free of selective reporting based on methods/results; x = selective reporting. 10. 10 ✓ = Free of other high bias risk; ? = insufficient data to determine risk of other bias. | | | | | | | | | | |

**Table S10.** Risk of bias for animal studies.
